# High-efficiency removal of cationic dye and heavy metal ions from aqueous solution using amino-functionalized graphene oxide, adsorption isotherms, kinetics studies, and mechanism

**DOI:** 10.55730/1300-0527.3462

**Published:** 2022-06-02

**Authors:** Metin ÇELEBİ, Eda GÖKIRMAK SÖĞÜT

**Affiliations:** Van Security School, Van Yüzüncü Yıl University, Van, Turkey

**Keywords:** Adsorption, amino functionalized graphene oxide, grapheneoxide, mechanism, Ni (II) ions, and rhodamine B dye

## Abstract

GO-NH_2_ (amino functionalized graphene oxide) was synthesized by grafting (3-aminopropyl) triethoxysilane onto the surface GO (graphene oxide). The GO-NH_2_ with a higher surface area and many active sites was characterized and its effectiveness in removing Rhod B dye and Ni(II) ions from wastewater by adsorption was observed. The effects of pH, time, and strange ions on the adsorption efficiency were investigated. The equilibrium isotherm data confirmed that it fits the Langmuir and Freundlich isotherm models. GO and GO-NH_2_ exhibited high adsorption capacity (Q_M_); 2500 (mg L^−1^) and 3333 (mg L^−1^) for Rhod B dye, and 312.5 (mg L^−1^), and 714.28 (mg L^−1^) for Ni(II) ions, respectively. The kinetics of adsorption was studied using pseudo-first-order, pseudo-second-order, intraparticle diffusion, and the Boyd model. It was found that the adsorption followed pseudo-second-order and film diffusion model are effective in this adsorption process. Adsorption mechanisms have been attributed to possible electrostatic attractions, hydrogen bonding, and interactions. In summary, the experimental results showed the synthesized GO and GO-NH_2_ would be promising adsorbents to remove aqueous solutions contaminated with Rhod B dye and Ni(II) ions.

## 1. Introduction

Heavy metal ions and organic dyes caused serious concern due to their general coexistence in various industrial wastewater [1]. Rhodamine B (Rhod B) dye and Ni (II) ions in many industries such as rubber, plastics, leather/textile processing plants, industries, electrical contacts, plastic, and battery production are released as waste [2,3]. The presence of these persistent pollutants poses a serious threat to anoxic conditions and human health by affecting the penetration of sunlight, which has a profound effect on photosynthetic activities and aquatic respiration [4]. Adsorption remains the most widely used method to eliminate these emerging threats [5,6]. The use of both raw and fabricated materials as different alternatives in the adsorption process keeps the interest in materials science warm, especially. An ideal adsorbent should have the ability to quickly and efficiently remove toxic pollutants from environments to a safe level [7]. Nanomaterials play an increasingly important role in removing dye and metal ions due to their higher specific surface area and sequestering properties [8,9]. Recently, various nanomaterials are at various stages of research and development, and carbon-based nanomaterials are one of the materials with potential applications in wastewater treatment systems [10].

Graphene and its derivatives have generally been considered highly effective adsorbents due to their ultra-high surface area and numerous surface groups. It is prone to functionalization with different oxygen-containing functional groups such as carboxyl and hydroxyl groups, which can significantly increase its adsorption. The π-π stacking and van der wall forces support the adsorption capacity and adsorption capacity of dyes and heavy metals [11,12]. However, there are some disadvantages direct use of GO for wastewater treatment [13]. Both the tendency to aggregate in the layer-by-layer mode due to strong inter-orbital interactions that reduce the adsorption efficiency and the relatively limited number of functional groups are insufficient to remove wastewater pollutants effectively. Also, the oxygen-containing functional groups of GO in coordination with pollutants are not very stable. Recently, to improve the adsorption performance of GO, many researchers have focused on using amino group compounds to functionalize it. Synthesized GO-NH_2_ has more functional groups, surfactant sites, selectivity and separation performance than GO. Thus, aggregation is prevented and the selectivity and adsorption capacity of GO against pollutants is increased. [14–17]. In this study, GO- NH_2_ was synthesized to remove pollutants from aqueous solutions and studied its effectiveness in adsorption.

This study attempts to investigate the efficacy of GO and GO-NH_2_ as an adsorbent for rhodamine B (Rhod B) and Ni(II) ions and their roles in the active site formation in aqueous solution media. Firstly, the synthesized adsorbents were characterized. Then, the optimum conditions were determined to investigate the adsorbent performance in removing rhodamine B (Rhod B) and Ni (II) ions from the aqueous solution and batch adsorption experiments. The equilibrium sorption data were applied to the Langmuir, Freundlich, and Dubinin-Radushkevich isotherm models. Kinetic models were used for evaluating the mechanism and behavior of rhodamine B (Rhod B) dye and Ni(II) ions adsorption onto GO and GO-NH_2_. Desorption results were obtained and a possible adsorption mechanism was evaluated.

## 2. Materials and methods

### 2.1. Synthesis of GO and GO-NH2 adsorbent

**Scheme 1.**
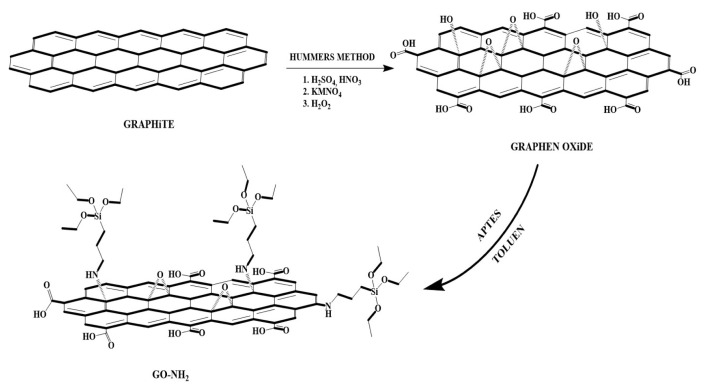
Synthesis protocol of GO and GO-NH_2_.

Graphene oxide (GO) synthesis: The Hummers Method was used in the synthesis of graphene oxide within the scope of this study (presented in Scheme 1). Five g of graphite, 115 mL of H_2_SO_4_ (98%), and 2.5 g of NaNO_3_ were mixed in an ice bath for 30 min without the temperature exceeding 20 °C. Fifteen g of KMnO_4_ was added portion-wise every 30 min to the mixture in the ice bath and stirred for one h. Then it was stirred at 45 °C for 1 h. After this time, 230 mL of distilled water was added and mixed rapidly for another 15 min. After adding 230 mL of water and stirring for 30 min at 45 °C, 600 mL of distilled water and 150 mL of H_2_O_2_ (9%) were added and mixed for 1 more h, and then washed with HCl (5%) solution prepared with filtered distilled water and dried.

Synthesis of amine functional graphene oxide (GO-NH_2_): One g of graphene oxide was taken into 100 mL of toluene and sonicated in an ultrasonic bath for 30 min. Then, 2.6 mL of 3-aminopropyltriethoxysilane (APTES) was added and stirred for 3 h at room temperature, then refluxed at 100 °C. After cooling to room temperature, the black product was filtered. Then, it was washed with toluene, distilled water and ethyl alcohol and dried under vacuum at 50 °C [18].

### 2.2. Sample characterization

GO examples were analyzed with DR-Uv/Vis (Shimadzu UV-2600 spectrophotometer), XRD (RIGAKU SMARTLABTM X-ray diffractometer) and SEM (scanning electron microscope-TESCAN MAIA3 XMU) and Raman spectra (Renishaw InVia Reflex Raman Microscopy System). Additionally, the FTIR spectra of the GO and GO-NH_2_ samples were taken on a Bio-Rad-Win-IR spectrophotometer carrying out KBr discs in between 4000 and 400 cm^−1^.

### 2.3. Preparation of RhB ve Ni (II) ions as adsorbate

**Figure 1:**
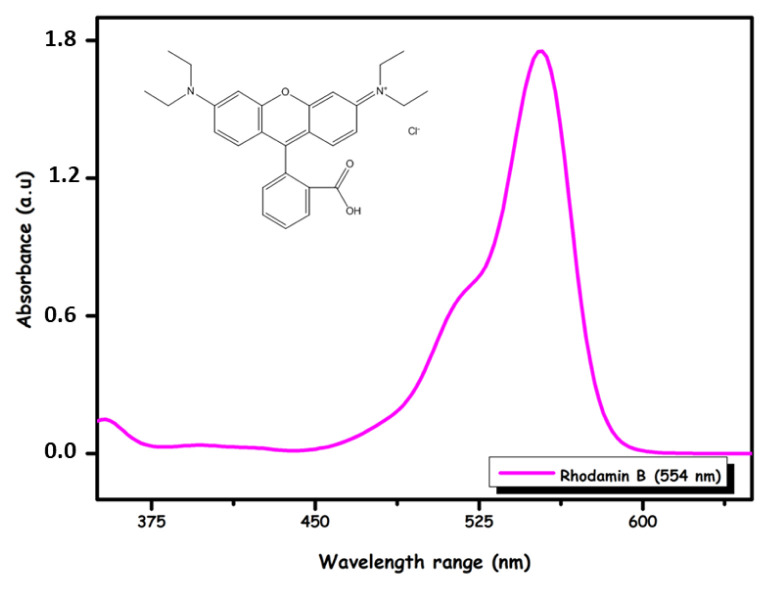
Structure and absorbance plots for Rhod B.

In this study, cationic synthetic rhodamine B dye with an acidic alligator (Rhod B) (C_28_H_31_CIN_2_O_3_; MA: 479.02 g mol-1; from Merck) is presented in Figure 1 and Ni(NO_3_)_2_ 6 H_2_O (MA: 290.79 g mol-1; from Merck) were used. Standard solutions (1000 mg L-1) were prepared to be diluted at different concentrations.

### 2.4. Adsorption experiments

Experiments were performed at 298 K using the standard batch adsorption procedure. The absorbance value of the remaining Rhod B dye solution was determined with a UV-vis spectrometer (Model Hitachi-2800), and the concentration of Ni(II) ions was determined using an AAS (atomic absorption spectroscopy) (Thermo Scientific ICE-3000). The calibration curve and absorbance of the dye concentration were determined to evaluate the amount of dye present in the adsorption samples. The absorbance of the Rhod B dye solution was measured on a Shimadzu UV-1800 spectrophotometer at a wavelength of 554 nm. The removal percentage (% R) was calculated using Equation (1) to evaluate the adsorption data.


(1) $$\%R=\frac{C_{0}-C_{e}}{C_{0}}\times 100\%$$

where C_0_ and C_e_ (mg L^−1^) are the initial and final concentrations of the Rhod B dye ve Ni (II) ions.

An equilibrium isotherm was used to demonstrate the removal efficiency of GO and GO-NH_2_ and evaluated from its expression (qe vs. Ce). The adsorption behavior of adsorbents to RhodB dye Ni(II) metal ions was studied in depth based on the theory of adsorption isotherm (Langmuir, Freundlich, Dubinin–Radushkevich) and kinetic models (pseudo-first-order, pseudo-second-order, intraparticle diffusion, Boyd plot) using Originlab 9.0 software. The results were evaluated, the applicability of the model and the correlation coefficient (R^2^) of their linear fit were compared. The linear equations of the three models are presented below [19].


(2) $$\frac{1}{q_{e}}=\frac{1}{K_{L}Q_{M}}.\frac{1}{C_{e}}+\frac{1}{Q_{M}}\;Langmuir$$

(3) $$log\;q_{e}=log\;K_{F}+\frac{1}{n}\;log\;C_{e}\;Freundlich$$

(4) $$\text{ln }q_{e}=\text{ln }q_{s}-k_{DR}\cdot {ɛ}^{2}\;Dubinin\text{-}Radushkevich$$

where C_e_ represents the equilibrium dye concentration (mg L^−1^); qe represents equilibrium and Q_M_ represents the maximum adsorption capacity (mg g^−1^); K_L_, K_F,_ and k_DR_ represent the constants Langmuir, Freundlich and DR, respectively. ɛ and n indicate Polanyi potential and adsorption density, respectively [20,21]. The equilibrium parameter (also known as the separation factor; R_L_) was used to examine whether the adsorption process was positive or negative for the Langmuir adsorption model and was derived using Equation 5.


(5) $$R_{L}=\frac{1}{1+K_{L}C_{0}}$$

where the adsorption isotherm is considered positive (0 < R_L_ <1), negative (R_L_ >1), linear (R_L_ =1) and irreversible (R_L_ =0), respectively, depending on the R value.


(6) $$E={\left(2k\right)}^{-\frac{1}{2}}$$

The value of the average adsorption energy (E) gives information about whether the adsorption process is physical or chemical. If the E value is below 8 kJ mol^−1^, the adsorption process is called physical adsorption; when the E value is between 8 and 16 kJ mol^−1^, the adsorption process is called ion exchange or chemical adsorption [22].

Kinetic studies were carried out to determine the adsorption rate of Rhod B dye and Ni(II) ion from an aqueous solution; pseudo-first-order and pseudo-second-order. In addition, the intraparticle diffusion model and Boyd model were used to evaluate the transport mechanism. Equations representing the mentioned models are presented below [23].


(7) $$\text{log}(q_{e}-q_{t})=\text{log }q_{e}-\frac{k_{t}}{2,303}\cdot t\;\;\;Pseudo-first\;order$$

(8) $$\frac{t}{q}=\frac{1}{k_{2}\cdot q_{e}^{2}}+\frac{1}{q_{e}}\cdot t\;Pseudo-second\;order$$

(9) $${\text{q}}_{\text{t}}={\text{k}}_{\text{i}}.{\text{t}}^{1/2}+\text{C }Intraparticle\;diffusion$$

(10) Bt=-0.4977-ln(1-F) Boyd model

(11) F=qtqe

where k_1_, k_2,_ and k_i_ represent pseudo-first-order, pseudo-second-order and intraparticle diffusion rate constant. q_e_ and q_t_ represent the amount of adsorption (mg g^−1^) at equilibrium and at any time t [24]. F represents the fraction of the adsorbate adsorbed at any time t, and B_t_ is a mathematical function of F [25].

### 2.5. Desorption experiments

It was performed using distilled water and HCl as the desorption agent. Desorption was investigated by contacting 10 mg of dye/metal-loaded adsorbent with 50 mL of 0.1 M HCl. The dye/metal loaded samples were desorbed using 0.1 M HCl, washed with distilled water, dried and used for the next adsorption cycle.

## 3. Results and discussion

### 3.1. Characterization of GO and GO-NH_2_

**Figure 2:**
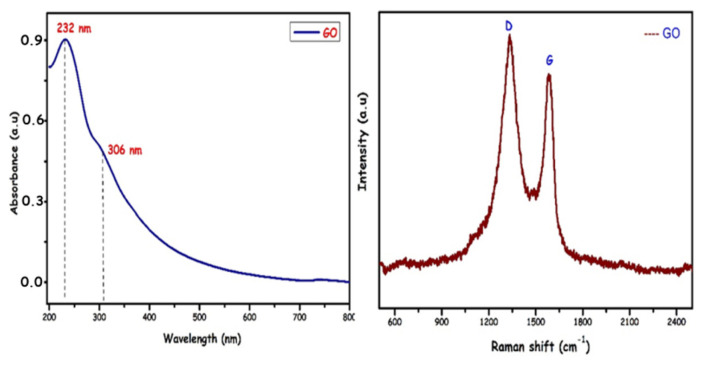
DR-Uv/Vis and Raman spectra of GO.

In Figure 2, the DR-UV/vis and Raman spectrum of GO is given. For GO, the π→π* transition of the aromatic C-C ring and the n→π* transition of the C=O bond is seen at 232 and 306 nm, respectively [26]. In the Raman spectrum of GO in the range of 2500–500 cm^−1^, characteristic D and G bands were observed at 1330 and 1580 cm^−1^, respectively [27].

**Figure 3:**
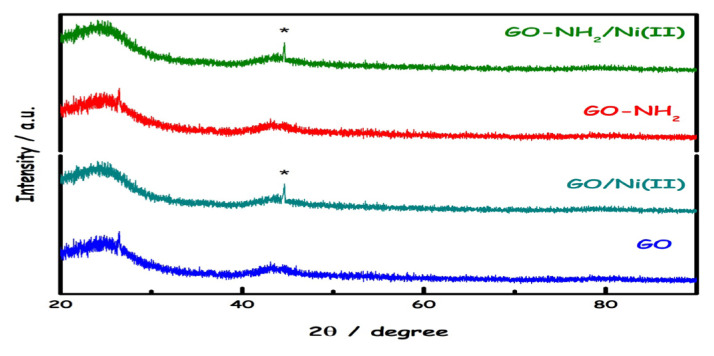
XRD patterns of Ni (II) ions before-after adsorption onto GO and GO-NH_2._

The broadening of the XRD spectra is due to the amorphous GO structure (Figure 3). In the obtained XRD spectra, characteristic peaks of the graphene oxide structure were observed at 23.8° and 43.1° as expected. In the XRD spectra of GO/Ni(II) and GO-NH_2_/Ni(II) structures, a prominent peak at (111) 44.9° assigned to the nickel ion was observed, but the characteristic peaks of nickel at 52° and 76° were not fully observed [28]. Since no significant change was observed in XRD intensities after dye adsorption, it was not evaluated.

**Figure 4:**
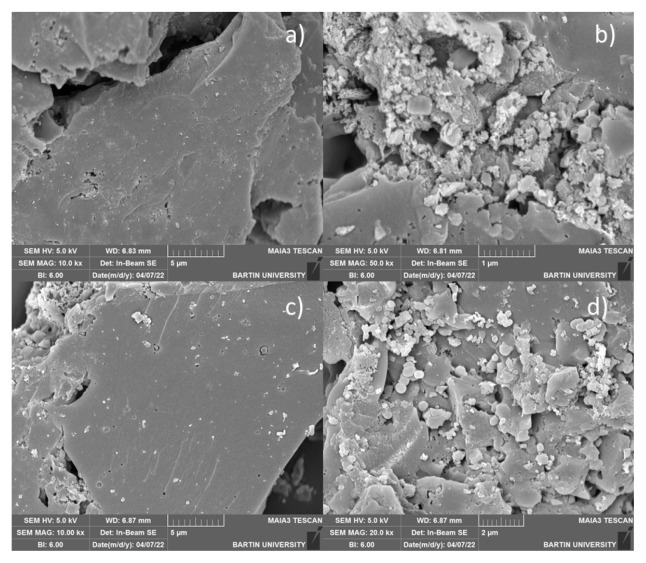
SEM images of Ni (II) ions before-after adsorption onto GO (a-b) and GO-NH_2_ (c-d).

As a result of the SEM images of the structures shown in Figure 4, the GO and GO-NH_2_ structures were found to be planar, and aggregation was observed upon adsorption of Ni(II) ions. Since no change was observed in the morphological structure after Rhod B dye adsorption, it was not evaluated.

**Figure 5:**
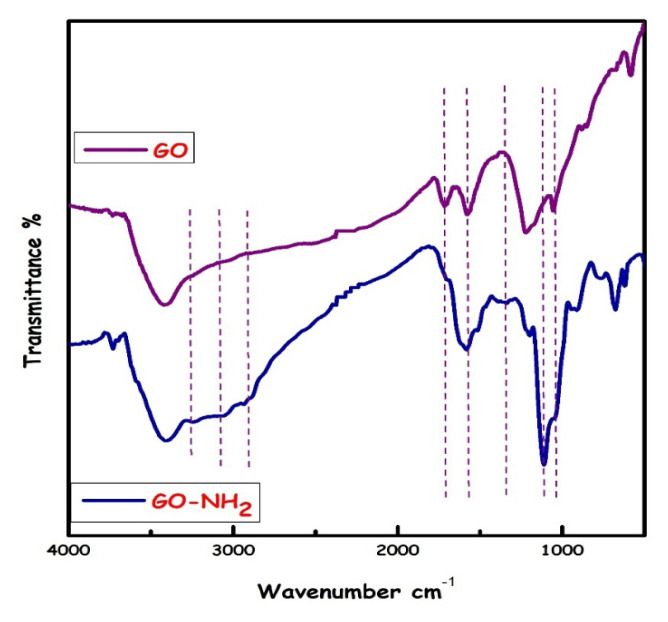
FTIR spectra of GO and GO-NH_2_.

In the FT-IR spectra given in Figure 5, the -OH vibrational band at 3451–3342 cm^−1^ in probable GO, C=O strong vibration band (1729–1707 cm^−1^), C=C vibration band (1617–1579 cm^−1^), CO (epoxy) vibration bands (1052–972 cm^−1^) were observed. The vibration band of –OH/-NH in GO-NH_2_ was observed at 3393–3219 cm^−1^, C-H vibration band (3051–2921 cm^−1^), weaker C=O vibration band (1696 cm^−1^) were observed. C=C vibration band (1586 cm^−1^), =C-H vibration band (1467–1389 cm^−^1), Si-O-Si strong vibration band (1112 cm^−1^) and Si-O-C vibration band (1046 cm^−1^) were seen as expected. The strong C=O vibration band in the spectrum of GO is weakly seen in GO-NH_2_; the presence of strong Si-O-Si and Si-O-C vibration bands instead of C-O (epoxy) groups in the GO structure supports that the surface of the GO structure is functionalized with 3-aminopropyltriethoxysilane. It is known that graphene sheets contain functional hydroxyl groups in their basal planes and edges. These groups react with 3-aminopropyltriethoxysilane (APTES) to obtain a GO-NH_2_ sample [29,30]. The functional -OH, -NH, -CO (epoxy), -C=O groups in the structure of GO-NH_2_ predicted that can make a hydrogen bond or electrostatic interaction with metal ions (Ni **^2+^**) or rhodamine B dye (-COOH/-N(CH_3_) groups).

### 3.2. Adsorption results

#### 3.2.1 Effect of contact time on the removal efficiency

**Figure 6:**
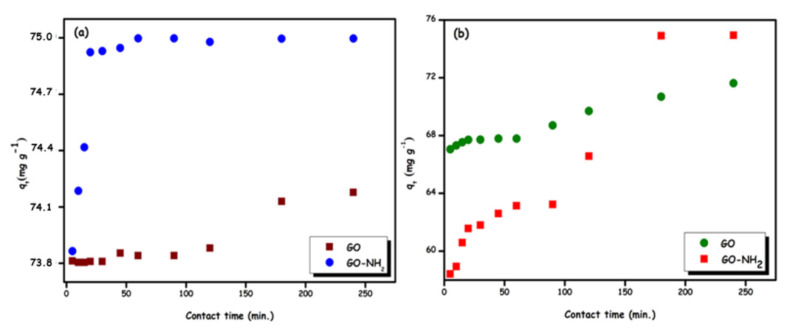
Effect of contact time (pH: 7; C_i_ = 30 mg L^−1^; T = 298 K) on the adsorption of Rhod B dye (a) and Ni (II) ions (b) onto GO and GO-NH_2_.

Observation of the adsorption time is an essential step in elucidating the adsorption process and obtaining information about the nature of the adsorbent. In Figure 6, it is observed that the Rhod B and Ni (II) ion adsorption on GO did not change much over time, while the adsorption on GO-NH_2_ first reached equilibrium after a rapid increase. The adsorption of Rhod B dye was observed that stabilized after 30 min. It demonstrates that the Rhod B dye adsorption rate on GO-NH_2_ is relatively high, and there are many easily accessible sites. However, the adsorption of Ni(II) ions, equilibrium was observed at 200 min. As a result, the adsorption equilibrium time had been determined at 200 min as the optimum condition.

#### 3.2.2 Effect of pH

The adsorption capacity of materials can be affected by the concentration of proton ions in an aqueous solution. Therefore, pH control is essential to capture the dye and metal ions in the adsorbate effectively. The optimum pH value also depends on the nature of the adsorbent used. For example, graphene oxide consists of various functional groups such as epoxy, hydroxyl, ketone, and carboxyl groups. When graphene oxide is dispersed in water, a negative charge is formed on its surface due to the ionization of its surface functional groups. While showing that the graphene oxide surface remains negatively charged in almost the entire pH range (pH_pzc_ value above 2) [31], GO-NH_2_ has a pH_pzc_ of 3.8 [32]. The GO-NH_2_ surface becomes negatively charged when the pH is higher than 3.8. In addition, the hydroxyl and carboxylic functional groups at the GO-NH_2_ edges develop negative charges due to deprotonation [32,33]. The interaction between GO and GO-NH_2_ adsorbents is a result of functional groups on the adsorbent surface and charges on dye molecules/heavy metal ions. Dyes are complex aromatic organic compounds with unsaturated bonds and various functional groups. Consequently, they have different ionization potentials at different pHs and cause a net charge change on the dye molecules [20].

**Figure 7:**
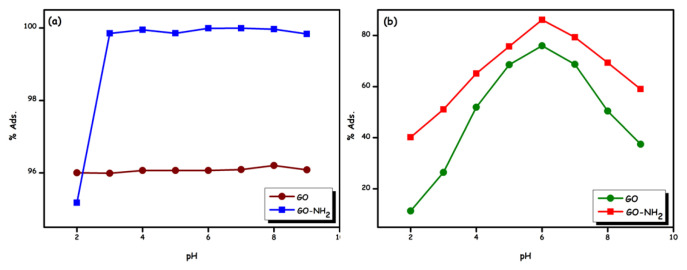
Effect of pH (C_i_ = 30 mg L^−1^; t: 200 min; T = 298 K) on the adsorption of Rhod B dye and Ni (II) ions onto GO and GO-NH_2_.

In Figures 7a–7b, the effect of pH (pH = 2–9) on the adsorption of Rhod B dye and Ni (II) ions on GO and GO-NH_2_ is presented. The pH of the solution affects the uptake of dye and heavy metal ions, as the surface charge of the adsorbent, the degree of ionization, and the type of adsorbate are affected. It is shown that solution pH significantly influences Ni (II) ions adsorption while it has little effect on rhodamine B adsorption. It was observed that the Rhod B dye removal, presented in Figure 7a, was largely unaffected by the increasing pH as pH increased from 2 to 9. Rhod B dye is a basic dye it contains a carboxyl group and the overall charge is positive. The reduction in Rhod B dye adsorption at pH 2 can be attributed to the electrostatic repulsion between the negatively charged surface and the Rhod B dye [34]. In addition, since the dye solution is not buffered, a change in the pH of the dye solution is expected during the sorption process. Further, while both the initial and equilibrium pH were initial pH = 4.83, the final pH was higher than the initial pH (6.58), indicating the possibility of either releasing OH^−^ ions into the solution or removing H^+^ ions from the solution [35].

The maximum adsorption removal of Ni(II) ions from GO and GO-NH_2_ aqueous solution occurred at a pH between 4.5–7 (in Figure 7). Therefore, pH 6 was accepted as the optimum pH for further studies. The effect of pH can be explained by considering the surface charge on the adsorption material. At low pH, due to the high positive charge density due to protons in the surface regions, electrostatic repulsion will be increased during the uptake of metal ions, resulting in lower removal efficiency. With increasing pH, electrostatic repulsion decreases due to a decrease in positive charge density. Adsorption sites thus result in increased metal adsorption. The above fact about the effect of pH on adsorption has been supported by several previous researchers [36–38]. At higher pH values, OH^−^ ions compete with active sites on the surface of adsorbents for Ni(II) [36]. Additionally, in the range above pH 8, nickel is present in the form of Ni(OH)^+^ [39].

#### 3.2.3 Effect of various ions

It contains various suspended and dissolved compounds that can be considered impurities in wastewater from the textile manufacturing or dyestuffs manufacturing industries. These impurities can be acids, alkalis, salts or metal ions. Cations such as Na^+^, K^+^, Cu^2+^, Ca^2+^, and Cr^3+^ are the most common ions found in dye-containing wastewater [40].

**Figure 8:**
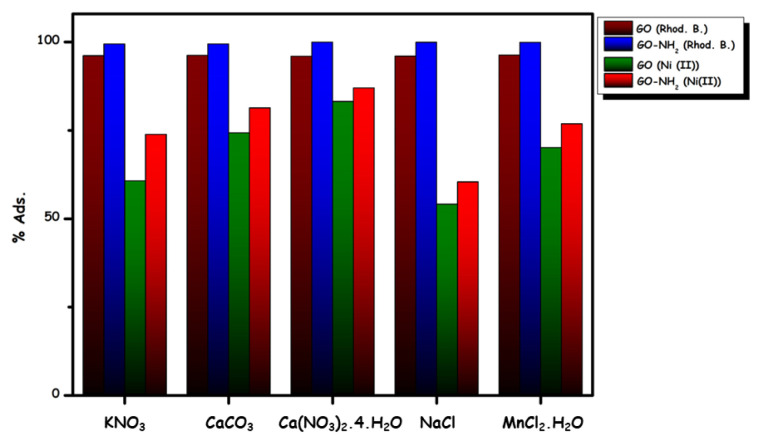
Effect of various ions for Rhod B dye (a) and Ni (II) ions adsorption (C_i_ = 0.3 M (strange ions); C_i_ = 30 mg L^−1^ (dye/metal ions); T = 298 K; pH = 6.5).

In Figure 8, the effect of different ions (KNO_3_, CaCO_3_, Ca(NO_3_)_2_.4H_2_O, NaCl and MnCl_2_.H_2_O) on the adsorption ability was observed. While the adsorption percentage does not decrease in Rhod B dye removal, a decrease is observed in Ni(II) ion removal. This decrease may be due to competition from other positively charged ions for the same binding sites on the adsorbent. Thus, the suitability of GO and GO-NH_2_ for the ion exchange mechanism is supported in working environments with different ions [41].

### 3.3. Adsorption isotherms

**Figure 9:**
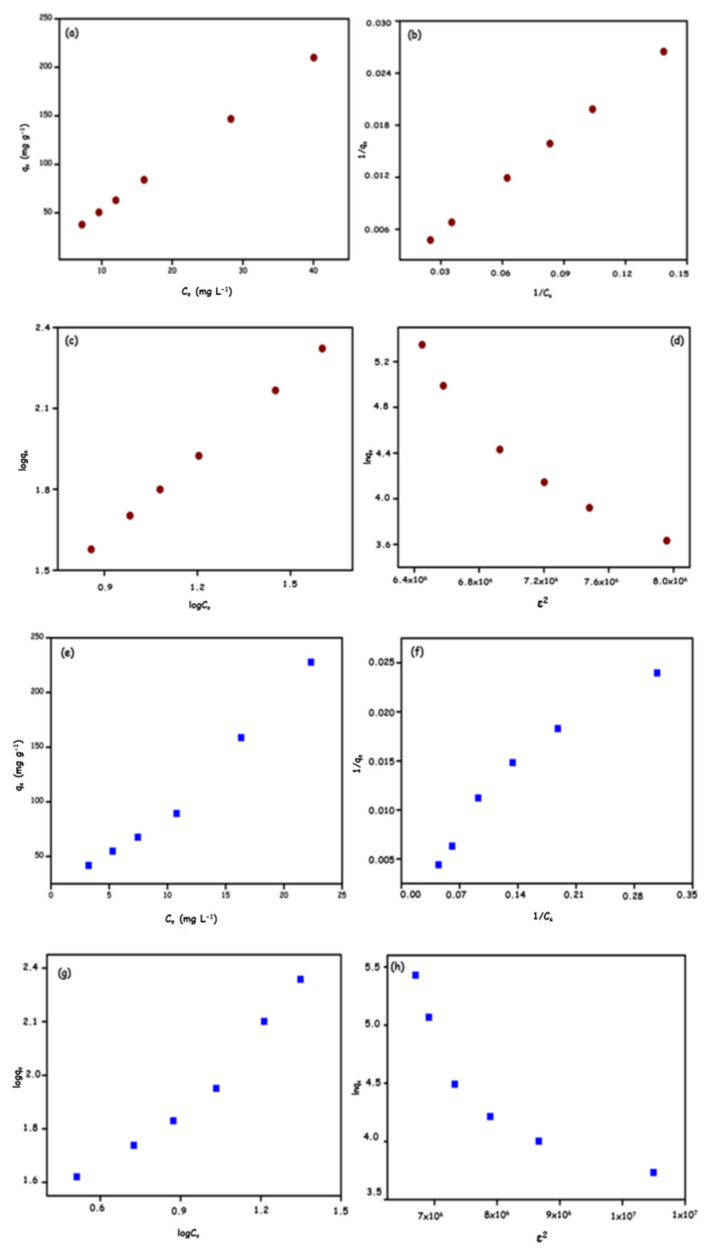

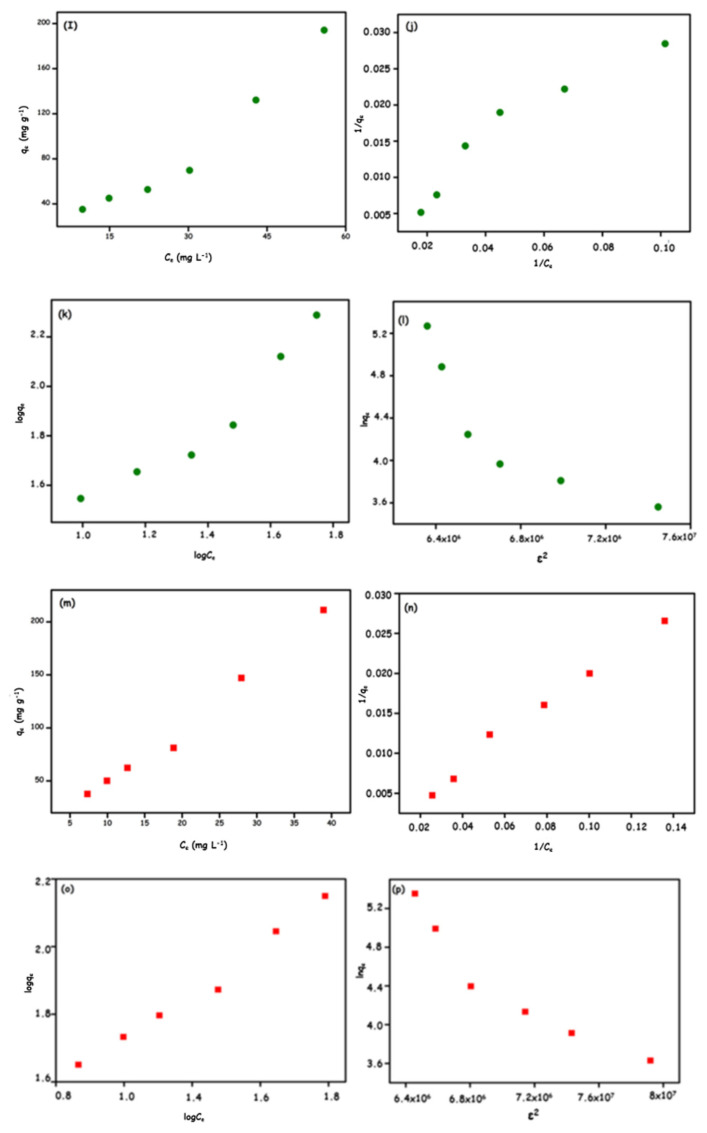
Adsorption isotherm models for Rhod B; GO (a-d):GO-NH_2_(e-h) and Ni(II) ions; GO (ı-l):GO-NH_2_(m-p).

The equilibrium isotherm is of fundamental importance for the design and optimization of the adsorption system and it is necessary to establish the optimum correlation for the equilibrium curves. Because of comparing the linear fit and correlation coefficients (R^2^) of the three models presented in Table 1 and Figure 9, it can be said that the Langmuir isotherm model fits better than the Freundlich and D-R isotherm models.

The comparison of the Q_M_ maximum adsorption capacity (mg g^−1^) followed in the following order; for Rhod B; GO-NH_2_ (3333 mg g^−1^) > GO (2500 mg g^−1^) > for Ni (II) ion; GO-NH_2_ (714.28 mg g^−1^)> GO (312.5 mg g^−1^). In the Freundlich isotherm model, the 1/n value obtained for all adsorbents is below 1, which indicates the appropriate adsorption condition. The adsorption energy (E) represents the energy required for moving one mole of solute from infinity to the surface of the adsorbent. The adsorption energies (E) obtained from the DR isotherm model range between 0.707–1.0018 kJ mol^−1^ at 298 K. Since E values are less than 8 kJ mol^−1^, the adsorption process of Rhod B and Ni (II) ions onto GO and GO-NH_2_ is physical.

### 3.4. Adsorption kinetics

**Figure 10:**
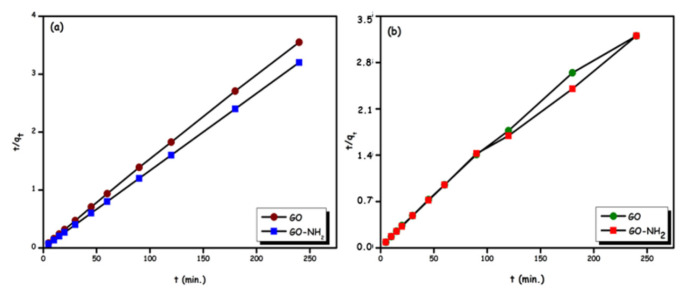
Adsorption kinetic models for Rhod B; GO and Go-NH_2_ (a) and Ni(II) ions; GO and Go-NH_2_ (b).

According to the correlation coefficients (R^2^) presented in Table 2 and Figure 10, the pseudo-second-order (PSO) kinetic model fits better. It was also found that the q_e(cal)_ values obtained from the pseudo-second-order (PSO) kinetic model were comparable to q_e(exp)_ compared to the pseudo-first-order (PFO) kinetic model. Since it could not be determined that the step controlling the adsorption rate from the PFO and PSO kinetic models was film or pore diffusion, the kinetic data were also analyzed and interpreted with the intraparticle diffusion model and the Boyd model [21].

**Figure 11:**
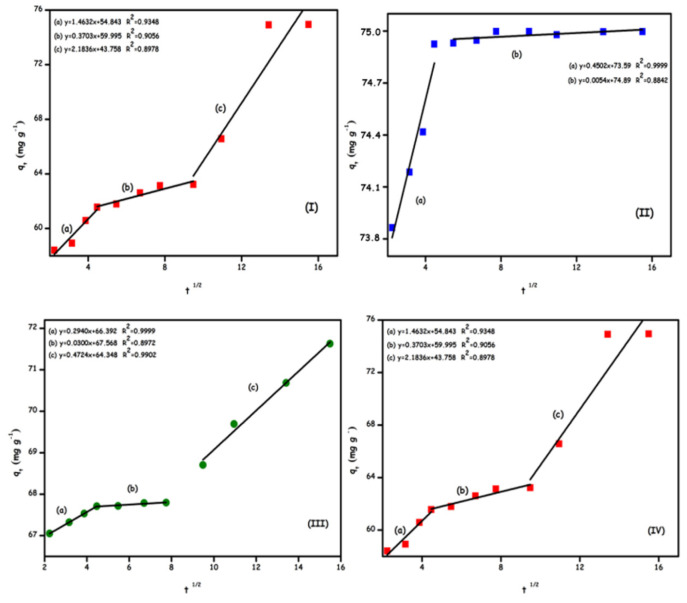
Intraparticle diffusion models for Rhod B; GO (a), Go-NH_2_ (b), and Ni(II) ions; GO (c) and Go-NH_2_ (d).

An adsorption process can be completed by either transduction, film or intraparticle diffusion (diffusion of the adsorbent within the pores) or film diffusion and intraparticle diffusion. The kinetic data were fitted to the intraparticle diffusion model and it was observed that the graph did not pass through the origin. It indicates that intraparticle diffusion was not the only rate-limiting step. According to the lines set in Figure 11, the first stage is the transport of adsorbate molecules to the outer surface of the adsorbent (film diffusion) and the second stage; represents the intraparticle diffusion of the adsorbent into its pores. In the third stage, the adsorbate was adsorbed to the inner region of the adsorbent [42]. The third stage was not considered a rate-limiting step as it is comparatively faster than the other two stages. It explains that this adsorption process fits this model well since most correlation coefficients (R^2^) were higher than 0.90. In Figure 9, Rhod B dye adsorption on GO-NH_2_ occurred in two steps; other adsorption processes occurred in three stages. A lower slope corresponds to a slower adsorption process. For this reason, it is concluded that the film diffusion steps were the rate-limiting step in the adsorption process [43].

**Figure 12:**
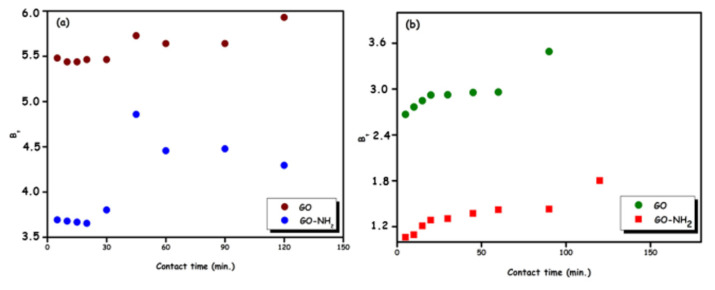
Boyd models for Rhod B; GO, GO-NH_2_ (a) and Ni(II) ions; GO, Go-NH_2_ (b).

Boyd plots with Bt versus t plot were obtained using Equations 9 and 10. A straight plot through the origin means that the adsorption process is controlled by intraparticle diffusion. Otherwise, the adsorption process is controlled by film diffusion or both film and intraparticle diffusion. From the experimental results, the plots of Boyd’s equation were nonlinear and did not pass through the origin (Figure 12). As a result, both models support that the rate-limiting step of the adsorption process is film diffusion [23,44].

### 3.6. Desorption

**Figure 13:**
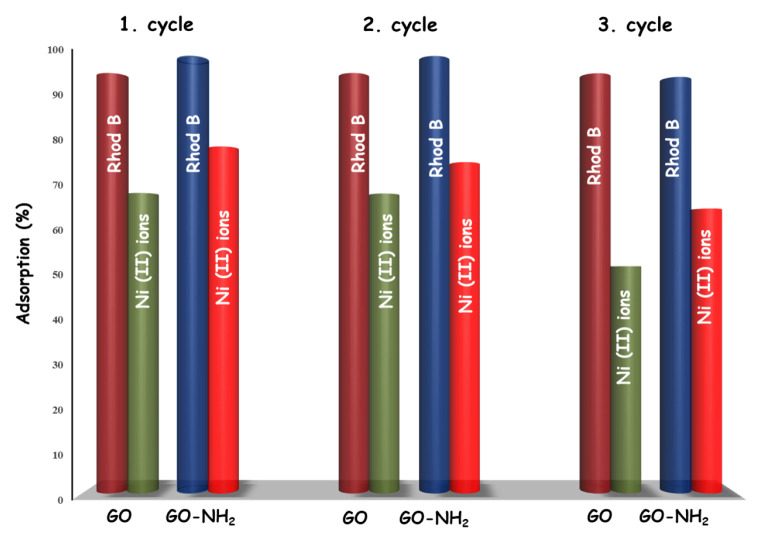
The performance of GO and GO-NH_2_ for three cycles of adsorption-regeneration for Rhod B dye and Ni (II) ions (pH = 7).

Desorption studies are essential to elucidate the nature of the adsorption process and to recover the adsorbent. Since the adsorption process’s economics depends on the adsorbents’ regeneration, its practical application is indispensable for industries [41]. As shown in Figure 13, the adsorption/desorption cycles were repeated three times. The results can conclude that although the adsorption capacity decreases slightly after each cycle, the synthesized GO and GO-NH_2_ can be used repeatedly with negligible loss of adsorption capacity for dye and metal ions.

### 3.7. Adsorption mechanism

**Figure 14:**
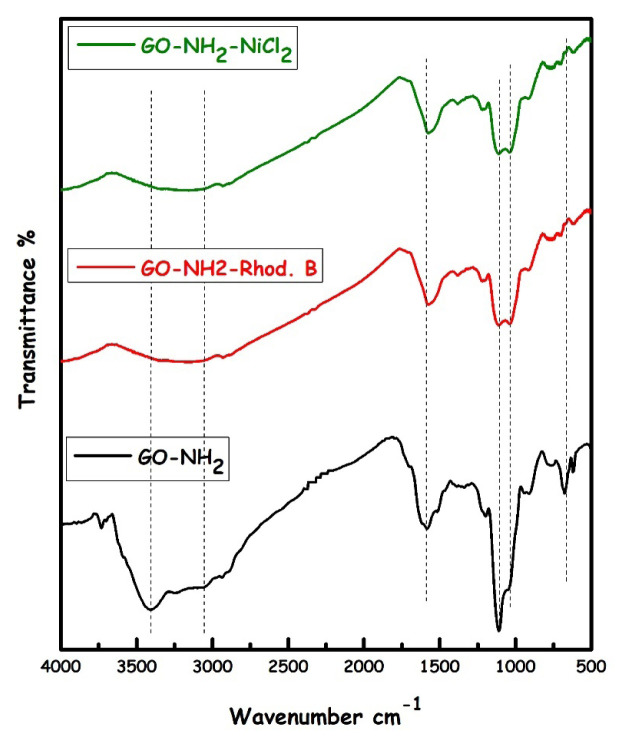
FTIR spectrum of GO-NH_2_, GO-NH_2_-NiCl_2_ and GO-NH_2_-Rhod B structures.

**Figure 15:**
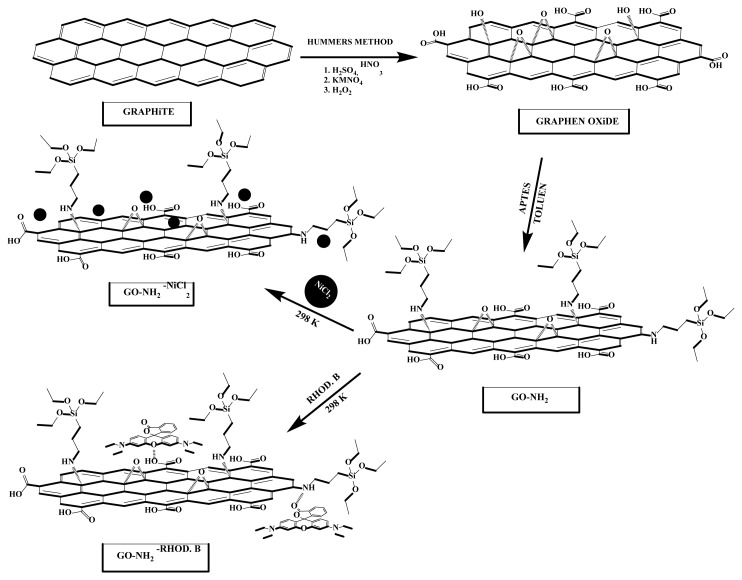
Schematic representation of Rhod B and Ni (II) ions on the GO, GO-NH_2_ surface.

Adsorption is a surface phenomenon in which adsorbates (ions or molecules) are held together by van der Waals forces (physisorption) or by forming chemical bonds (chemical adsorption) [45]. Also, adsorption mechanisms resulting from the electrostatic interaction, hydrogen bonding and π-π interaction have been reported as the primary mechanisms for graphene-based adsorbents to remove organic dyes [46]. Interestingly, various types of forces such as H-bonds, electrostatic interactions, ion exchange and π-π interactions are responsible for physical adsorption [47]. For example, GO carries many functional groups, such as carboxylic acids, that can readily release protons in water and cause an adsorbent surface to charge. In this case, the adsorption is probably governed by electrostatic interactions. Since GO-NH_2_ has various oxygen-containing active functional groups such as hydroxyl, carbonyl and epoxy groups, it can be adsorbed via (a) ion exchange, (b) surface complexation, and (c) electrostatic attraction [32]. The decrease in the intensity of the ν(C=O) peak and the broadening of the ν(OH) peak in the FTIR spectrum (Figure 14) also confirm the binding of these functional groups via electrostatic attraction and H-bond. Moreover, the adsorption process might occur due to the π–π stacking interaction of the aromatic ring of the rhodamine B dye with the nanolayers of GO samples. In this study, physisorption can be attributed to the adsorption of dye (Rhod B) and heavy metal (Ni (II) ions) ions on GO and GO-NH_2_ (Figure 15).

### 3.8. Comparison with other adsorbents

In Table 3, the adsorption capacities of other adsorbents used to remove Rhod B dye and Ni (II) ions from an aqueous solution were compared. After functionalizing both adsorbents with high efficiency, especially with the -NH_2_ group, their active areas increased and their performance improved.

## 4. Conclusions

In this study, GO and amino-functionalized GO (GO-NH_2_) were synthesized by the Hummers method to investigate the adsorption properties of Rhod B dye and Ni (II) ions. Maximum adsorption capacities of Rhod B dye and Ni (II) ion on GO and GO-NH_2_ are 2500, 3333, 312.5, and 714.28 mg g^−1^ at optimal pH = 6 values, respectively, the adsorption kinetics agreed well with the pseudo-second-order kinetic model. The sorption isotherms are compatible with the Langmuir model. The maximum removal efficiency (99.9%) was obtained for Rhod B dye in industrial wastewater with GO-NH_2_. The adsorption-desorption results showed that the reusability of GO and GO-NH_2_ is encouraging and effective in dye and metal removal. The easy separation of Rhod B dye and Ni (II) ions from charged GO and GO-NH_2_ samples using HCl acid solution shows that the adsorption mechanism could be explained by physisorption.

## Figures and Tables

**Table 1:** Adsorption isotherm parameters.

Isotherm models		Rhod B		Ni(II)	
		GO	GO-NH_2_	GO	GO-NH_2_
Langmuir	Q_M_ (mg g^−1^)	2500	3333	312.5	714.28
	K_L_	0.0013	0.0003	0.0012	0.0007
	R_L_	0.9447	0.9867	0.9488	0.9694
	R^2^	0.9999	0.9899	0.9101	0.9895
Freundlich	K_F_	1.8958	1.0018	3.1318	2.1967
	1/n	0.9969	0.9803	0.9730	0.9569
	R^2^	0.9999	0.9844	0.9214	0.9855
Dubinin-	q_M_ (mg g^−1^)	2189	2464	178.3	393.03
Radushkevich	K_D-R_	1.E-6	5.E-7	1.E-6	1.E-6
	E_D-R_	0.707	1.001	0.756	0.707
	R^2^	0.9374	0.8551	0.7495	0.8903

**Table 2:** Value of constants of various kinetic models.

Kinetic		Rhodamine B		Ni (II) ions	
Models		GO	GO-NH_2_	GO	GO-NH_2_
	**q** **_e (exp)_** ** (mg g** **^−1^** **)**	**67.6269**	**74.7501**	**68.4926**	**7.4903**
Pseudo-first order	q_e. (cal)_ (mg g-1)	2.4216	2.8511	6.3489	4.9843
	k_1_ (min-1)	0.0145	0.0070	0.7121	0.0689
	R^2^	0.9794	0.3426	0.5285	0.8743
Pseudo-second order	q_e. (cal)_ (mg g−1)	66.6660	75.1880	73.5294	75.7576
	k_2_ (g mg^−1^ min−1)	0.0122	0.2211	0.0022	0.0020
	R^2^	1.0000	1.0000	0.9947	0.9957

**Table 3:** Comparison of different adsorbents for removal of Rhod B. dye and Ni (II) ions.

Adsorbent	Adsorbate	Adsorption capacity (q_M_)	Isotherms	Ref.
Waste seeds	Rhod. B	117 (mg g−1)	Sip	[2]thermogravimetric analysis (TGA
Aleurites moluccana (WAM)				
ZSM-5 Synthesized from Bangka kaolin	Rhod. B	128.21 (mg g−1)	Langmuir	[48]
Activated carbon	Rhod. B	255.39 (mg g−1)	Langmuir	[49]
UiO-66-(COOH)2	Rhod. B	2200 (mg g−1)	Langmuir	[50]
GO-NH_2_	Rhod. B	3333 (mg g−1)	Langmuir and Freundlich	This study
Nano kaolinite	Ni(II) ions	111 (mg g−1)	Langmuir	[51]
Rice husk (microwave-functionalized cellulose)	Ni(II) ions	80.38 (mg g−1)	Langmuir	[52]microwave-functionalized cellulose derived from rice husk was cost-effectively prepared and employed for Pb(II
Hydrogel	Ni(II) ions	286.7 (mg g−1)	Langmuir	[53]
Chitosan (CS) (CS-g-AOPAM)	Ni(II) ions	213.4 (mg g−1)	Langmuir	[54]
GO-NH_2_	Ni(II) ions	714.28 (mg g−1)	Langmuir and Freundlich	This study
